# Cultivation of Botany in Ohio

**Published:** 1833

**Authors:** 


### Art. X. Cultivation of Botany in Ohio.

We take great pleasure in acknowledging the receipt of a port-folio of Ohio plants, collected, beautifully preserved, and accurately labelled, by Mr. Riddell, of the valley of the Scioto; to which they are indigenous. We hope that gentleman will continue his researches, especially into the herbaceous botany of our prairies and diluvial formations, with their lakes and morasses. Those tracts unquestionably produce many plants, not yet known to the science; and it would afford us great pleasure to make our Journal the vehicle of their publication.

